# Prevalence of Cyclomodulin-Positive *E. coli* and *Klebsiella* spp. Strains in Mexican Patients with Colon Diseases and Antimicrobial Resistance

**DOI:** 10.3390/pathogens11010014

**Published:** 2021-12-23

**Authors:** Adrian Canizalez-Roman, Juan E. Reina-Reyes, Uriel A. Angulo-Zamudio, Eloy E. Geminiano-Martínez, Antonio F. Flores-Carrillo, Rolando R. García-Matus, Norma M. Valencia-Mijares, Nidia Leon-Sicairos, Jorge Velazquez-Roman, Francisco A. Martínez-Villa, Gabriela Tapia-Pastrana

**Affiliations:** 1 Centro de Investigación Aplicada a la Salud Pública (CIASaP), School of Medicine, Autonomous University of Sinaloa, Culiacan Sinaloa 80246, Mexico; canizalez@uas.edu.mx (A.C.-R.); uriel.zamudio@uas.edu.mx (U.A.A.-Z.); nidialeon@uas.edu.mx (N.L.-S.); jorgevelazquezroman@uas.edu.mx (J.V.-R.); 2 The Women’s Hospital, Secretariat of Health, Culiacan Sinaloa 80127, Mexico; 3 Laboratorio de Investigación Biomédica, Hospital Regional de Alta Especialidad de Oaxaca, San Bartolo Coyotepec, Oaxaca City 71256, Mexico; jerr_230390@hotmail.com (J.E.R.-R.); enriquegeminiano@yahoo.com.mx (E.E.G.-M.); dr.antoniofc79@gmail.com (A.F.F.-C.); rolandogarciamatus@yahoo.com.mx (R.R.G.-M.); valencia_norma18@yahoo.com.mx (N.M.V.-M.); 4 Facultad de Medicina y Cirugía, Universidad Autónoma Benito Juárez de Oaxaca, Oaxaca City 68120, Mexico; 5 Pediatric Hospital of Sinaloa, Constitución 530, Jorge Almada, Culiacan Sinaloa 80200, Mexico; 6 Programa de Maestría en Ciencias en Biomedicina Molecular, Facultad de Medicina, UAS, Culiacan Sinaloa 80246, Mexico; tps48_@hotmail.com

**Keywords:** cyclomodulins, colon diseases, *E. coli*, DEC, *Klebsiella* spp.

## Abstract

Colon diseases, such as colorectal cancer (CRC), are multifactor diseases that affect more than one million people per year; recently, the microbiota has been associated with an etiologic factor, specifically bacterial cyclomodulin positivity (CM^+^). Unfortunately, there are no studies from Mexico that detail the presence of bacterial CM^+^ in patients with colon diseases. We therefore performed a comprehensive study to investigate the associations and prevalence of cyclomodulin-positive Diarrheagenic *E. coli* (DEC)*,* non-DEC, and *Klebsiella* spp. strains isolated from Mexican subjects with colon diseases. In this work, we analyzed 43 biopsies, 87 different bacteria were isolated, and *E. coli* was the most frequently noted, followed by *Klebsiella* spp., and *Enterococcus* spp. *E. coli*, non-DEC, and EPEC belonging to phylogroup B2 were the most prevalent. More than 80% of *E. coli* and *Klebsiella* were CM^+^. *pks*, *cdt*, *cnf*, and *cif* were identified. *cdt* was associated with non-DEC, *cif* and its combinations with EPEC, as well as *cdt* and *psk* with *Klebsiella*. Lastly, all the CM^+^ bacteria were resistant to at least one antibiotic (34% were MDR, and 48% XDR). In conclusion, the high prevalence of bacterial CM^+^ in colon disease patients suggests that these bacteria play an important role in the genesis of these diseases.

## 1. Introduction

Colon diseases are a common complex of diseases in humans ranging from inflammatory bowel disease (IBD) to colorectal cancer (CRC). Cancer is the abnormal growth of cells in determinate tissue, and is the result of multiple alterations in the cell cycle [1]. CRC is the third most common malignancy, and fourth most common cause of cancer mortality worldwide; in fact, it is estimated that there are more than 1 million new cases of CRC each year [2], and CRC is responsible for 896,000 deaths per year worldwide [3]. The process of CRC starts with chronic inflammation in the gut, and then the patient progresses to colitis. After that, DNA damage and mutations in oncogenes and tumor suppressor genes occur in cells in the intestine. These phenomena lead to the production of polypi or diverticula, and the constant damage to DNA will evolve into CRC [4,5].

There are many factors associated with CRC, such as diet, lifestyle, genetic predisposition, and, recently, the microbiota [6]. Evidence shows that the composition of the human intestinal microbiota influences the host health status. In fact, microbial dysbiosis has been observed in CRC patients, and there is evidence that infection from bacteria, including *Streptococcus bovis*, *Enterococcus* spp., *Helicobacter pylori*, *Bacteroides fragilis*, *Escherichia coli*, and *Klebsiella* spp., can result in CRC [7]. Some studies have demonstrated a high presence of *E. coli* in colon diseases in comparison with controls. Studies have revealed that from 70% of CRC biopsies, *E. coli* has been isolated in comparison with healthy subjects [8]. Interestingly, most *E. coli* isolated from colon diseases belong to phylogroup B2; of all the *E. coli* phylogroups, B2 is related to strong adherent-invasive ability and high virulence skills that are needed to cause damage to the host [9]. Other bacteria that form part of the normal microbiota and have been isolated from CRC biopsies are *K. pneumoniae*; this bacterium has been isolated from 13.5% of CRC patients in comparison with healthy subjects [10].

*E. coli* and *Klebsiella* are Gram-negative bacteria that colonize in humans, starting from the first hours of life. These bacteria are a component of normal colon-rectal flora; however, cyclomodulins have been identified in colon-rectal flora isolated from colon diseases. Cyclomodulins are genotoxins that modulate the cellular cycle, differentiation, apoptosis, and proliferation [7]. Different cyclomodulins have been identified, some of which are (i) colibactin, which is in the *pks* genomic island, and causes DNA double-strand breaks and chromosomal instability in human eukaryotic cells [11]; (ii) cytotoxic necrotizing factor (CNF), which activates Rho GTPases, leading to cytoskeletal alterations, and affecting the cell cycle [12]; (iii) cycle-inhibiting factor (CIF), which is related to proliferation inhibition by mitosis inhibition, blocking the cell cycle G2/M transition [13]; and (iv) cytolethal distending toxin (CDT), which apparently induces DNA damage by DNAse activity [14]. The presence of cyclomodulin-positive bacteria in biopsies of colon diseases suggests that these bacteria play an important role in the genesis of these diseases.

Despite the importance of these bacterial cyclomodulins in colon diseases, there are no studies about the prevalence of these bacteria in Mexican subjects with colon diseases. Therefore, in this comprehensive study, we investigated the associations and prevalence of cyclomodulins-positive (*pks*, *cdt*, *cnf*, and *cif*) diarrheagenic *E. coli* (DEC), non-DEC, and *Klebsiella* spp. strains isolated from Mexican subjects with colon diseases (IBD, polypus, diverticulosis, and CRC).

## 2. Results

### 2.1. E. coli, Klebsiella, and Enterococcus in Colon Disease Biopsies

From 43 biopsies, 87 different bacteria were isolated: 32 bacteria from CRC, 18 from polypus, 13 from IBD, and 5 from diverticulosis patients (Table 1). Although commensal bacteria, such as *E. coli*, are not commonly isolated from lesions (i.e., colon biopsy tissue) in the gut, *E. coli* was the most frequent bacteria isolated from colon disease biopsies (60.9%), followed by *Klebsiella* (19.5%), *Enterococcus* (18.4%), and the combination of *E. coli* + *Enterococcus* (1.1%). Regarding biopsies from CRC, *E. coli* was the most prevalent bacterium (53.1%) compared to the rest of the colon disease biopsies, but without significant associations, whereas *Enterococcus* was associated with CRC biopsies, being the second most prevalent (28.1%, Table 1). Notably, the combination of *E. coli* + *Enterococcus* was found only in CRC biopsies. *Klebsiella* was the most prevalent polypus biopsy (27.8%) after *E. coli,* and the same distribution was observed in IBD and diverticulosis biopsies without significant associations (Table 1).

### 2.2. Pathotypes and Phylogroups of E. coli Isolated from Colon Disease Biopsies

To identify and classify the pathotypes and phylogroups of *E. coli*, we performed PCR assays. Regarding the pathotypes, we identified EPEC and DAEC, and the rest of the *E. coli* strains were classified as non-DEC. Of the 53 *E. coli* strains isolated in this study, non-DEC was the most prevalent (49.0%), followed by EPEC (45.2%) and DAEC (5.6%) (Table 2). Non-DEC was the most prevalent in polypus (58.3%) and IBD (57.8%) biopsies, whereas EPEC was most frequently observed in CRC (58.8%) and diverticulosis (60%) biopsies. DAEC was present only in CRC (5.8%) and IBD (10.5%) biopsies (Table 2). Nevertheless, no statistical (*p* > 0.05) associations were found among the *E. coli* pathotypes and colon disease biopsies.

Regarding the *E. coli* phylogroups, B2 was the most prevalent (41.5%), followed by B1 (32.1%), and A and D in lower proportions (13.2%, Table 3). With respect to the pathotypes and phylogroups, EPEC (63.6%) was the most frequently noted in B2, with a high presence in polypus biopsies (35.7%). In B1, non-DEC was the most prevalent (47.1%) from the IBD biopsies (50%). In phylogroup D, EPEC was dominant (57.1%) of those isolated from CRC biopsies (75%), and lastly, in phylogenetic group A, we identified only non-DECs, and those isolated from CRC were the most prevalent (42.8%) (Table 3). However, no statistical associations were found among *E. coli* phylogroups and colon disease biopsies.

### 2.3. Distribution of Cyclomodulins (pks, cnf, cdt, and cif) in E. coli and Klebsiella spp. Isolated from Colon Disease Biopsies

The distribution of CM-encoding genes from the *E. coli* and *Klebsiella* strains is shown in Table 4, Table 5 and Table 6. First, we evaluated the strains isolated from colon disease biopsies to see if they were positive or negative for CM genes (Table 4). Overall, 80.4% of the strains were CM^+^, whereas 19.5% were negative. Regarding *E. coli*, this bacterium was statistically associated with the presence of CM in comparison with the negative strains (75.7% vs. 0%). *Klebsiella* also presented a statistically significant association with cyclomodulins (24.3% vs. 0%). However, all the *Enterococcus* strains were negative for cyclomodulins (Table 4).

We then evaluated the distribution of individual CM genes (*pks*, *cnf*, *cdt,* and *cif*; Table 5). Of the total CM^+^ strains, non-DECs represented 37.1%, EPECs reached 34.2%, DAECs 4.2%, and *Klebsiella* 24.8%. The CM gene most prevalent in *E. coli* and *Klebsiella* strains was *cdt* (35.7%), followed by *pks* (20%), *cnf* (14.2%), and *cif* (4.2%). Additionally, we found different combinations of CM genes, such as *cnf/cdt* (8.5%), *cnf-cif* (5.7%), *cdt*/*cif* (2.8%), *cdt*/*pks* (2.8%), and *cnf*/*cdt*/*cif* (5.7%, Table 5). Regarding the associations among the strains (DEC or non-DEC, and *Klebsiella)* and CM genes, statistical associations were found between non-DEC and *cdt* (*p* < 0.001); EPEC was associated with *cif, cnf/cif,* and *cnf/cdt/cif* (*p* < 0.001); and *Klebsiella* was associated with *pks* and *cdt* (*p* < 0.001) (Table 5).

The relationship between strains encoding CM genes and colon disease biopsies was also analyzed (Table 6). The *pks*^+^ strains were isolated in a higher proportion from patients with polypus. The *cnf*^+^ strains were more prevalent in IBD biopsies, whereas the *cdt*^+^ and *cif*^+^ strains were more prevalent in patients with CRC (Table 6). Strains encoding CM genes in combination presented a homogenous distribution among colon disease biopsies (Table 6). Despite the trend in the data, no statistical (*p* > 0.05) associations were found among strains encoding CM genes and colon disease biopsies.

### 2.4. Antimicrobial Resistance of the E. coli and Klebsiella spp. Isolated from Colon Disease Biopsies

The antibiotic resistance of the bacteria isolated from the biopsies is shown in Table 7. Overall, bacteria presented high resistance to antibiotics tested. More than 50% of non-DEC strains presented a high resistance to ampicillin, piperacillin, amoxicillin/clavulanic acid, cefazolin, streptomycin, and nalidixic acid antibiotics, whereas more than 50% of EPEC were resistant to ampicillin, piperacillin, cefazolin, cefotaxime, ceftriaxone, streptomycin, tetracycline, ciprofloxacin, levofloxacin, and nalidixic acid (Table 7). More than 50% of DAEC were also resistance to ampicillin, amoxicillin/clavulanic acid, cefazolin, ceftriaxone, imipenem, and streptomycin. In *Klebsiella* spp., a high resistance to ampicillin, piperacillin, cefazolin, streptomycin, and nalidixic acid was presented (Table 7). It is important to mention than most of the bacteria were susceptible to chloramphenicol. When we compared the antibiotic resistance among all bacteria, we found that DAEC was more resistant to imipenem (*p* = 0.02), whereas *Klebsiella* spp. were more resistant to gentamicin and tobramycin (*p* = 0.04 and 0.03, respectively), EPEC was more resistant to tetracycline and levoflaxin (*p* = 0.0005 and 0.0012, respectively), and the other bacteria had similar resistant profiles. Concerning strains classified as resistant, 34.3% were MDR, and 48.6% were XDR in the comparison among bacteria, and they presented a homogenous distribution without significant associations, whereas in MDR strains, DAEC (66.7%) and *Klebsiella* (58.8%) were significantly more prevalent than the rest of the strains. For XDR, EPEC (75%) presented a higher prevalence (Table 7).

After analyzing the antibiotic resistance of strains isolated from colon disease biopsies, we searched for a relation between the presence of CM genes and antibiotic resistance (Table 8). All the CM^+^ strains were resistant to antibiotics, and only 4.3% of strains were resistant to one antibiotic; *cdt*^+^ (4%), *cif*^+^ (33.3%), and the combination *cnf/cdt*^+^ (25%) were resistant to one antibiotic. Interestingly, 95.7% of the CM^+^ strains were resistant to more than two antibiotics (*p* < 0.001), of which 100% of strains positive for *pks, cnf, cnf/cif, cdt/pks,* and *cnf/cdt/cif* were included in this category, as well as 96% positive for *cdt*, 66.6% positive for *cif*, and 75% positive for *cnf/cdt* (Table 8). It is quite clear that the CM^+^ strains presented high resistance to antibiotics.

## 3. Discussion

In this study, we demonstrated that *E. coli* (non-DEC, EPEC, and DAEC) from phylogroup B2 was the most prevalent bacterium isolated from the colon disease biopsies (CRC, polypus, diverticulosis, and IBD), followed by *Klebsiella* and *Enterococcus*. A total of 80.4% of the isolated strains were CM^+^, of which non-DEC was statically associated with *cdt*, EPEC with *cif* and its combinations, whereas *Klebsiella* was associated with *pks* and *cdt*. Lastly, 34.3% of the isolated strains presented the MDR phenotype, and 48.6% presented XDR; surprisingly, more than 95% of the CM^+^ strains were resistant to two or more antibiotics.

*E. coli* and *Klebsiella* are classified as important commensal bacteria in the human gut, but recently, these bacteria have been isolated from lesions in the gut related to colon diseases. In a previous work, a prevalence of 39.5% to 12.9% of *E. coli* in colon disease biopsies, such as CRC and diverticulosis [8], was found, whereas *K. pneumoniae* was found in 4–27% of patients with CRC [15,16], data that are similar to those found in this study. Other bacteria isolated from colon diseases in this study were *Enterococcus*, which was associated with CRC patients. The presence of this bacterium in CRC has also been reported by other researchers, but its mechanism of producing damage is still unclear [17].

Different *E. coli* pathotypes (EPEC and DAEC) were found in patients with colonic diseases in this study, with EPEC being the most prevalent. The presence of EPEC has previously been associated with CRC. It has reported an EPEC prevalence of 50.7% in CRC patients in Egypt compared with 20.1% in healthy patients, and this prevalence was higher than that found in the Mexican population [18]. Prorok-Hamon et al. (2014) found a high prevalence of DAEC *afaC*^+^ and *lpfA^+^* in CRC and IBD patients in comparison with that in controls [19]. Both pathotypes were considered in this study due to their strong ability to adhere to enterocytes, which is the first step in producing damage in cells. In the case of EPEC, this pathotype harbors *eae* (*E. coli* attachment effacement), which helps to form attaching-and-effacing (A/E) lesions on intestinal cells, and contains a type 3 secretion system [20,21], whereas DAEC encodes important adhesins, such as afimbrial (*Afa*) or fimbrial (*Dr*) adhesins [22].

Regarding the *E. coli* phylogroups, most of the *E. coli* strains isolated in this study belonged to B2, and these data match those of other studies. In a work, it was reported that 47.5% and 32.6% of *E. coli* isolated from CRC and diverticulosis patients, respectively, belonged to phylogroup B2 [9]. Also, 66.4% *E. coli* B2 prevalence was found in biopsies of patients with colon diseases (CRC and diverticulosis) in French subjects [8]. This *E. coli* is frequently found in colon diseases because it is well documented that B2 *E. coli* have a greater ability to colonize the human intestine, as this bacterium encodes fitness factors, such as pili and adhesins [23]. Despite the methodology applied to clean the biopsies and remove the mucus surface, *E. coli* remained in the samples, which demonstrated the high capacity of *E. coli* B2 to attach to the surface; nevertheless, the probability of bacteria internalized into enterocytes cannot be discarded. Moreover, *E. coli* belonging to phylogroup B2 has been related to the presence of cyclomodulins, which are responsible for causing damage to the DNA of host cells, and altering the cellular cycle [24].

Cyclomodulins have been identified in different bacteria, such as *E. coli* or *Klebsiella,* that were isolated from colon diseases. The prevalence of bacterial cyclomodulin positivity in colon diseases differs depending on the population. In this study, approximately 80% of the bacteria were cyclomodulin-positive, which was higher than that in other studies, such as that in French subjects, in which 52.3% of *E. coli* strains from patients with colon diseases were positive for this genotoxin [24]. Other work reported an *E. coli* cyclomodulin positivity of 66.7% in CRC patients, and 40% in patients with IBD [25]. Also, the presence of 25.6% cyclomodulin-positive *K. pneumoniae* was observed [15]. The high prevalence of bacteria positive for cyclomodulins in colon diseases, specifically in lesion zones, suggests that these bacteria have a role in the development of colon diseases. In the case of *pks*-encoded colibactin, this genotoxin causes DNA double-strand breaks and chromosomal instability in human eukaryotic cells, and its relation to CRC is well established [11]. Previous reports indicate that *pks* is most frequently found in *E. coli* isolated from patients with CRC [8]. By contrast, in our study, we found that this cyclomodulin was associated with *Klebsiella* spp., and was found in similar proportions in all colon diseases from IBD to CRC, suggesting that *pks* could cause damage to DNA during the early steps of colon diseases. Additionally, based on the *K. pneumonia pks*^+^ prevalence shown in this study, it is important to increase the research focus on this bacterium as an etiologic agent of colon diseases. Moreover, the high presence of bacterial *pks^+^* in this study, as in other studies, demonstrated the importance of this genotoxin in the development of colon diseases.

*cnf* is a cyclomodulin that promotes cellular proliferation by stimulating the assembly of actin stress fibers and focal adhesions by deamination and DNA synthesis, and *cnf* has only been found in *E. coli* and *Y. pseudotuberculosis*. Although *cnf* has been associated with uropathogenic *E. coli* and bladder cancer, in this study, *cnf* was found in DEC and non-DEC, and primarily in CRC patients [26]. A previous report detected *cnf-1* in stool samples for the first time, but only one sample was positive for this genotoxin from healthy people in Puerto Rico. In contrast, in our study, *cnf^+^ E. coli* was isolated from 10 individuals. The high prevalence of *cnf* in our study in comparison with that in the Gómez-Moreno study could have occurred because our patients presented colon diseases, whereas the Gómez-Moreno study also included healthy participants [27].

Another important cyclomodulin found in this study was *cdt*, which has phosphodiesterase activity that triggers a DNA damage response resulting in inhibited proliferation by G2–M cell cycle arrest [12]. There are different types of CDT toxins, and these genes are within the chromosomal DNA, such as *cdtA* or *cdtB*, and the other is in plasmid DNA, such as *cdt-II*. CDTs have also been described in other important bacterial pathogens, such as in *E. coli* strains, but to date, still not yet in *Klebsiella* spp. or isolated from patients with colon diseases. This is the first study to report the presence of *cdt* in *Klebsiella* spp.

The active interchange of genetic information (competence or recombination process) in the gut among bacteria could be the factor that helps *Klebsiella* spp. acquire this genotoxin, and this fact is very important because bacteria not only disseminate among the genes that confer antibiotic resistance, but also virulence factors, which can be watersheds in the virulence of bacteria [28,29]. However, future research is needed to characterize the structure and function of this *cdt* in *Klebsiella* spp. and compare it with the *cdt* of other bacterial species, and also identify the *Klebsiella* species by biochemical and molecular methods in this way to know what *Klebsiella* species is positive to *pks* or *cdt*. Notably, *cdt* was the most prevalent cyclomodulin in this study; despite the relationship between *cdt* and DNA damage, this toxin has also been related to serious cases of diarrhea in Mexican subjects due to *E. coli* [30,31]. However, we found an association of *cdt* with non-DECs, but recent work indicates that non-DECs with supplementary virulence genes have high levels of virulence, and are capable of producing diarrhea [32]. The high prevalence of diarrheas from *E. coli* in Mexico could explain the amount of *cdt* in our study population [33].

Regarding *cif*, this genotoxin is related to proliferation inhibition by mitosis inhibition, which blocks the cell cycle G2/M transition [13]. In this study, the cycle-inhibiting factor was found only in *E. coli*, specifically in EPEC, because *cif* must secrete type III into the system to translocate to the cytoplasm of the host cells, where it induces a progressive cytopathic effect [34]. This cyclomodulin was less prevalent in Mexican subjects, which is consistent with other studies [8]. This is the first study to identify *E. coli* with a combination of three cyclomodulins. In a previous study, *E. coli* with a combination of two cyclomodulins (*cnf/cdtdf* and *cnf/pks*) was isolated [8].

The high antibiotic resistance of strains isolated from patients with colon diseases in this study is alarming. *E. coli* and *Klebsiella* spp. were resistant to multiple antibiotics, as observed in other similar studies in Mexico [32,35,36]. A study conducted in Egypt also reported a high resistance to antibiotics in *E. coli* and in *K. pneumoniae* isolated from patients with CRC, where more than 71% of those strains were resistant to 10 antibiotics. [37]. However, other studies have reported low or moderate resistance of *E. coli* and *Klebsiella* positive for cyclomodulins to antibiotics [38,39]. More evidence is needed to establish a possible relationship between antibiotic resistance and cyclomodulins; nevertheless, we cannot rule out the resistance of the present microbiome in subjects from other countries. In the case of Mexico, bacteria isolated from humans, water, or foods have a high level of resistance to antimicrobials [32,35,36].

To the best of our knowledge, this is the first paper to report the presence of cyclomodulins in Mexican subjects. We also highlighted that this is the first time that *cdt* has been identified and associated with *Klebsiella* spp. Lastly, we demonstrated that cyclomodulin-positive bacteria have high resistance to antibiotics, and that one bacterium can harbor up to three cyclomodulin genes.

In conclusion, this study provides evidence regarding the high prevalence of cyclomodulin-positive bacteria in patients with colon disease in Mexican subjects. *E. coli* belonging to phylogroup B2 was the most frequently isolated from patients. Some cyclomodulins were associated with different bacteria, and no difference was found among cyclomodulins and colon diseases. Moreover, high antibiotic resistance was present in CM-positive bacteria. The findings presented in this study support that the presence of bacteria positive for these genotoxins may have a role in the development of colon diseases, and special attention should be given to these bacteria as etiologic agents of colon diseases. In this context, new strategies must be established to detect and eradicate bacteria harboring the CM gene, and prevent the development of colon diseases.

## 4. Material and Methods

### 4.1. Ethical Considerations

This research project was approved by the Committee of Ethics in Research and the Research Committee of Regional Hospital of High Specialty of Oaxaca (HRAEO) under registration number HRAEO-CIC-CEI-015-16. The patients were informed, and we asked for their participation in the protocol. They were informed about the protocol, procedures, and risks of sampling. We voluntarily obtained written consent from the patients, and they were informed that they could withdraw their consent at any time.

### 4.2. Patients

In this study, 43 adult patients of both sexes were recruited from February 2015 to May 2017 from the HRAEO. Sixteen patients had CRC, nine had polypus, thirteen had inflammatory bowel disease, and five had diverticulosis. The age range of patients with cancer was 35 to 84 years (median 57), the age of diverticula patients ranged from 32 to 72 years (median 58), the age of polypus patients ranged from 20 to 77 years (median 54), and the age of inflammatory bowel disease patients ranged from 39 to 84 years (median 50.5).

The sex proportion (men/women) was 1.6 in CRC patients, 3.5 in diverticula patients, 0.22 in polypus patients, and 5 in patients with inflammation. Biopsies were taken by the HRAEO medical team from injured or inflamed tissue. For the colonoscopy, patients fasted for 8 h, and they took polyethylene glycol or sodium picosulfate to prepare their intestines for the procedure.

### 4.3. Bacteria Strains

The reference strains used in this study belong to our laboratory collection. To identify DEC, we used DAEC (daaE+), ETEC (lt+ and/or st+), EPEC E2348/69 (eae+ and bfp+), and enteroaggregative *E. coli* (EAEC O42; pCVD432+ and aafII+). *Klebsiella pneumoniae* subsp. pneumoniae (Schroeter) Trevisan ATCC 13883, ATCC 13182 was used to identify *Klebsiella*. *Enterococcus faecalis* ATCC 19433 to *Enterococcus*. *E. coli* (Migula) Castellani and Chalmers ATCC, ATCC BAA-2452, ATCC 25922, and ATCC 35218 were used as control in antimicrobial susceptibility tests. *E. coli* (Migula) Castellani and Chalmers ATCC 11775 and *E. coli* K12 (HB101) were used in quality control of culture media. The bacteria were cultured in Luria-Bertani (LB) broth at 37 °C, and frozen in 15% glycerol at −70 °C for future experiments.

### 4.4. Bacterial Isolation and Identification from Biopsies

Biopsies from colon mucosa were collocated in tubes containing sterile phosphate saline solution (PBS), and the biopsies were washed three times to clean the fecal waste. To obtain bacteria from the biopsies, Triton X-100 (0.1X) was added to the samples, and processed using a Tissue Ruptor II (Qiagen, Valencia, CA, USA). To identify bacteria in biopsies, we performed biochemical and molecular analyses. First, crushed biopsies were inoculated into selective agar culture media, namely, McConkey (MCD LAB, Oaxaca, Mexico), CHROMagar Orientation Medium (BD BBL, Franklin Lakes, NJ, USA), and *Enterococcus* Agar (BD BBL, Franklin Lakes, NJ, USA), to identify bacteria, such as *E. coli, Klebsiella* spp., and *Enterococcus* spp. These samples were incubated at 37 °C for 24 h. The colonies that grew in selective media were collected, and every colony was characteristic for each bacterium. To complete the biochemical analysis, the colony identities were confirmed using an API 20 E kit test (bioMérieux, Marcy l’Etoile, France) according to the manufacturer’s instructions. Twelve colonies were selected per sample, which were cultivated in LB broth, and frozen in 15% glycerol at −70 °C for future experiments.

### 4.5. Preparation of Template DNA

To obtain genomic DNA, 10 colonies were taken per sample, and inoculated into LB broth media incubated at 37 °C for 18 h. Each bacterial pellet was collected and suspended in 200 µL of molecular biology grade water, and then the boiling method was applied [32]. The DNA was stored at −20 °C for future experiments.

### 4.6. Molecular Identification of Bacterial Species, Pathotypes, Phylogenetic Groups, and Cyclomodulins

To complete the identification of the bacteria isolated from the biopsies, PCRs were performed, and the 16 S rRNA gene was amplified to confirm the identities of *E. coli*, *Klebsiella*, and *Enterococcus faecalis*. The primers used in the reaction are shown in Supplemental Table S1. Using the *E. coli* isolate strains, the following pathotypes were searched: enteropathogenic *E. coli* (EPEC) and diffusely adherent *E. coli* (DAEC), by identifying the *bfp* and *eae* genes in EPEC and *daaE* genes in DAEC (Supplemental Table S1). Bacteria that were negative for both reactions were classified as non-DEC, and the positive ones were denoted as DEC. Then, phylogenetic groups A, B1, B2, and D were identified as described by Clermont et al. [40]. Additionally, the identification of cyclomodulins CNF, CDT, CIF, and PKS was performed using the genes *cnf, cif, cdt III* and *IV*, *pks*, and *clbB* as targets. The primer sequences and the sizes of the PCR products are shown with these targets in Supplemental Table S1. For all PCR reactions, we mixed MyTaq (BIOLINE), 0.5 µM forward and reverse primers, and 2 µL of genomic DNA and molecular biology grade water, and the reactions were performed in a Mastercycler flexlid thermocycler (Eppendorf). The PCR products were visualized using ethidium bromide (1 µg/µL), and the results were analyzed in a PhotoDoc-It imaging system with a Benchtop 2 UV transilluminator (UVP).

### 4.7. Antimicrobial Agent Susceptibility Testing

Antibiotic susceptibility testing of the isolated bacteria was performed using the Kirby-Bauer disk diffusion method on Mueller-Hinton agar plates [41] in accordance with the guidelines of the Clinical Laboratory Standard Institute (CLSI) [42]. Suspensions of isolated bacteria were prepared in LB at a turbidity of 0.5 using the McFarland turbidity standard. Then, they were swabbed on already prepared nutrient agar plates. The plates containing the impregnated antibiotic disks (BD BBL, Franklin Lakes, NJ, USA) were placed aseptically on the inoculated agar. The tested antibiotics were ampicillin, piperacillin, amoxicillin, ampicillin/sulbactam, cefazolin, cefotaxime, cefuroxime, ceftazidime, aztreonam, imipenem, gentamicin, tobramycin, amikacin, kanamycin, streptomycin, tetracycline, ciprofloxacin, levofloxacin, nalidixic acid, and chloramphenicol, and the plates were incubated for 18 h at 37 °C. *E. coli* (Migula) Castellani and Chalmers (ATCC^®^ 25922™) was used as the control. After that, the diameters of the inhibition halos were measured by using the Vernier rule. Antibiotic susceptibility was interpreted according to CLSI guidelines; the bacteria were classified as resistant, intermediate, or sensitive. Isolates that showed non-susceptibility to at least one agent in more than three and six antibiotic categories were classified as multidrug-resistant (MDR) and extremely drug-resistant (XDR), respectively [43]. The antibiotics were selected based on their use in treating human infections caused by Gram-negative bacteria [44], and represent different classes of antimicrobial agents that are available to treat these infections in Mexico.

### 4.8. Statistical Analysis

Associations between nominal variables were analyzed with Fisher’s exact test and/or chi-squared test. Statistical significance was determined when *p* ≤ 0.05, and analyses were performed with the IBM^®^ SPSS^®^ Statistics program version 20 (New York, NY, USA).

## Figures and Tables

**Table 1:** Distribution of isolated bacteria from colon disease biopsies.

		Colon Diseases *n* (%)				
		CRC	Polypus	IBD	Diverticulosis	*p* Value
	Total Biopsies *n* = 43	*n* = 16	*n* = 9	*n* = 13	*n* = 5	
Isolated bacteria	*n* = 87 (%)	*n* = 32 (%)	*n* = 18 (%)	*n* = 29 (%)	*n* = 8 (%)	-
*E. coli*	53 (60.9)	17 (53.1)	12 (66.7)	19 (65.5)	5 (62.5)	0.87
*Klebsiella* spp.	17 (19.5)	5 (15.6)	5 (27.8)	4 (13.8)	3 (37.5)	0.34
*Enterococcus* spp.	16 (18.4)	9 (28.1)	1 (5.6)	6 (20.7)	0 (0.0)	0.03
*E. coli + Enterococcus*	1 (1.1)	1 (3.1)	0 (0.0)	0 (0.0)	0 (0.0)	0.62

CRC: colorectal cancer, IBD: inflammatory bowel disease. Fisher’s exact test was used to investigate statistical significance.

**Table 2:** Non-DEC, EPEC, and DAEC isolated from colon disease biopsies.

		Colon Diseases *n* (%)				
		CRC	Polypus	IBD	Diverticulosis	*p* Value
	Total Biopsies *n* = 43	*n* = 16	*n* = 9	*n* = 13	*n* = 5	
Isolated bacteria	*n* = 87 (%)	32 (36.8)	18 (20.6)	29 (33.4)	8 (9.2)	-
*E. coli* strains	53 (60.9)	17 (53.1)	12 (66.7)	19 (65.5)	5 (62.5)	0.87
Subtypes						
Non-DEC	26 (49.5)	6 (35.2)	7 (58.33)	11 (57.8)	2 (40.0)	0.46
EPEC	24 (45.2)	10 (58.8)	5 (41.6)	6 (31.5)	3 (60.0)	0.72
DAEC	3 (5.6)	1 (5.8)	0 (0.0)	2 (10.52)	0 (0.0)	0.57

CRC: colorectal cancer, IBD: inflammatory bowel disease. Fisher’s exact test was used to investigate statistical significance.

**Table 3:** *E. coli* phylogroups (A, B1, B2, and D) of strains isolated from colon disease biopsies.

	*E. coli* Pathotypes *n* = 53 (%)							
	A *n* = 7 (13.2)	B1 *n* = 17 (32.1)		B2 *n* = 22 (41.5)			D *n* = 7 (13.2)	
Colon Disease Biopsies	Non-DEC	Non-DEC	EPEC	DAEC	Non-DEC	EPEC	Non-DEC	EPEC
	*n* = 7 (100)	*n* = 8 (47.1)	*n* = 6 (35.3)	*n* = 3 (17.6)	*n* = 8 (36.4)	*n* = 14 (63.6)	*n* = 3 (42.9)	*n* = 4 (57.1)
CRC	3 (42.8)	1 (12.5)	3 (50.0)	1 (33.3)	1 (12.3	4 (28.6)	1 (33.3)	3 (75.0)
Diverticulosis	1 (14.2)	0 (0.0)	0 (0.0)	0 (0.0)	1 (12.3)	2 (14.3)	0 (0.0)	1 (25.0)
Polypus	1 (14.2)	3 (37.5)	0 (0.0)	0 (0.0)	2 (25.0)	5 (35.7)	1 (33.3)	0 (0.0)
IBD	2 (28.5)	4 (50.0)	3 (50.0)	2 (66.6)	4 (50.0)	3 (21.4)	1 (33.3)	0 (0.0)

CRC: colorectal cancer, IBD: inflammatory bowel disease.

**Table 4:** *E. coli* and *Klebsiella* containing CM-encoding genes that were isolated from colon disease biopsies.

		Cyclomodulins *n* (%)		
Isolated Bacteria	Total *n* = 87 (%)	Positive	Negative	*p* Value
		*n* = 70 (80.4)	*n* = 17 (19.5)	
*E. coli*	53 (60.9)	53 (75.7)	0 (0.0)	<0.001 *
*Klebsiella* spp.	17 (19.5)	17 (24.3)	0 (0.0)	0.03 *
*Enterococcus*	16 (18.3)	0 (0.0)	16 (94.1)	<0.001 *
*E. coli + Enterococcus*	1 (1.1)	0 (0.0)	1 (5.9)	0.19

Fisher’s exact test was used to investigate statistical significance. * *p* ˂ 0.05.

**Table 5:** Relation among bacteria isolated from biopsies of colon diseases and cyclomodulins.

Bacteria	Total Strains CM^+^ *n* = 70 (%)	Cyclomodulins *n* = 70 (%)								
		*pks*	*cnf*	*cdt*	*cif*	*cnf*-*cdt*	*cnf-cif*	*cdt-cif*	*cdt-pks*	*cnf-cdt-cif*
		*n* = 14 (20.0)	*n* = 10 (14.2)	*n* = 25 (35.7)	*n* = 3 (4.2)	*n* = 6 (8.5)	*n* = 4 (5.7)	*n* = 2 (2.8)	*n* = 2 (2.8)	*n* = 4 (5.7)
Non-DEC	26 (37.1)	5 (35.7)	4 (40.0)	11 (44.0) *	0 (0.0)	4 (66.7)	0 (0.0)	0 (0.0)	2 (100)	0 (0.0)
EPEC	24 (34.2)	0 (0.0)	4 (40.0)	5 (20.0)	3 (100) *	2 (33.3)	4 (100) *	2 (100)	0 (0.0)	4 (100) *
DAEC	3 (4.2)	0 (0.0)	2 (20.0)	1 (4.0)	0 (0.0)	0 (0.0)	0 (0.0)	0 (0.0)	0 (0.0)	0 (0.0)
*Klebsiella* spp.	17 (24.8)	9 (64.3) *	0 (0.0)	8 (32.0) *	0 (0.0)	0 (0.0)	0 (0.0)	0 (0.0)	0 (0.0)	0 (0.0)

Fisher’s exact test was used to investigate statistical significance. * *p* ˂ 0.05.

**Table 6:** CM-encoding genes of *E. coli* and Klebsiella isolated from patients with colon disease.

Colon Diseases Biopsies	*pks n* = 14 (%)		*cnfn* = 10 (%)			*cdt n* = 25 (%)				*cifn* = 3 (%)	*cnf/cdt n* = 6 (%)			*cnf/cifn* = 4 (%)	*cdt/cif n* = 2 (%)	*pks/cdt n* = 2 (%)	*cnf/cdt/cif n* = 4 (%)
	*Klebsiella*	Non-DEC	Non-DEC	EPEC	DAEC	*Klebsiella*	Non-DEC	EPEC	DAEC	EPEC	Non-DEC	EPEC	DAEC	EPEC	EPEC	Non-DEC	EPEC
	*n* = 9 (64)	*n* = 5 (36)	*n* = 4 (40)	*n* = 4 (40)	*n* = 2 (20)	*n* = 8 (33)	*n* = 11 (45)	*n* = 5 (21)	*n* = 1 (4)	*n* = 3 (100)	*n* = 2 (33)	*n* = 3 (50)	*n* = 1 (17)	*n* = 4 (100)	*n* = 2 (100)	*n* = 2 (100)	*n* = 4 (100)
CRC	2 (22.2)	1 (20.0)	1 (25.0)	1 (25.0)	1 (50.0)	3 (37.5)	3 (27.2)	1 (20.0)	NCD	2 (66.6)	1 (50.0)	1 (33.3)	NCD	2 (50.0)	1 (50.0)	NCD	NCD
Diverticulosis	2 (22.2)	NCD	NCD	2 (50.0)	NCD	1 (12.5)	1 (9.1)	1 (20.0)	NCD	NCD	NCD	NCD	NCD	NCD	NCD	1 (50.0)	1 (25.0)
Polypus	3 (33.3)	2 (40.0)	NCD	NCD	NCD	2 (25.0)	4 (36.3)	2 (40.0)	NCD	NCD	NCD	1 (33.3)	NCD	2 (50.0)	NCD	1 (50.0)	2 (50.0)
IBD	2 (22.2)	2 (40.0)	3 (75.0)	1 (25.0)	1 (50.0)	2 (25.0)	3 (27.2)	1 (20.0)	1 (100)	1 (33.3)	1 (50.0)	1 (33.3)	1 (100)	NCD	1 (50.0)	0 (0.0)	1 (25.0)

CRC: colorectal cancer, IBD: inflammatory bowel disease, NCD: No cyclomodulin detected.

**Table 7:** Antibiotic resistances of strains isolated from biopsies of colons.

Antibiotic	Resistant to *n* Antibiotics	Total Strains Isolated *n* = 70 (%)	*E. coli* Non-DEC *n* = 26 (%)	DEC		*Klebsiella* spp.	*p* Value
				*n* = 27 (%)		*n* = 17 (%)	
				EPEC *n* = 24	DAEC *n* = 3		
Ampicillin		58 (83.0)	20 (76.9)	20 (83.3)	3 (100)	15 (88.2)	0.65
Piperacillin		37 (52.8)	12 (46.1)	14 (58.3)	0 (0.0)	11 (64.7)	0.16
Amoxicillin/clavulanic acid		35 (50.0)	14 (53.8)	11 (45.8)	3 (100)	7 (41.1)	0.27
Ampicillin sulbactam		17 (24.2)	5 (19.2)	7 (29.1)	1 (33.3)	4 (23.5)	0.81
Cefazolin		68 (97.4)	24 (92.3)	24 (100)	3 (100)	17 (100)	0.32
Cefotaxime		34 (48.5)	12 (46.1)	14 (58.3)	1 (33.3)	7 (41.1)	0.65
Ceftriaxone		34 (48.5)	12 (46.1)	14 (58.3)	2 (66.6)	6 (35.2)	0.46
Ceftazidime		21 (30.0)	6 (23.0)	11 (45.8)	0 (0.0)	4 (23.5)	0.16
Aztreonam		24 (34.2)	9 (34.6)	11 (45.8)	0 (0.0)	4 (23.5)	0.27
Imipenem		11 (15.7)	1 (3.8)	5 (20.8)	2 (66.6)	3 (17.6)	0.02 *
Gentamicin		16 (22.8)	2 (7.6)	7 (29.1)	0 (0.0)	7 (41.1)	0.04 *
Tobramycin		20 (28.5)	4 (15.3)	7 (29.1)	0 (0.0)	9 (52.9)	0.03 *
Kanamycin		18 (25.7)	3 (11.5)	9 (37.5)	0 (0.0)	6 (35.2)	0.09
Streptomycin		58 (82.2)	20 (76.9)	20 (83.3)	2 (66.6)	16 (94.2)	0.43
Tetracycline		37 (52.8)	10 (38.4)	21 (87.5)	1 (33.3)	5 (29.4)	0.0005 *
Ciprofloxacin		25 (35.7)	7 (26.9)	13 (54.1)	0 (0.0)	5 (29.4)	0.09
Levoflaxin		26 (37.1)	8 (30.7)	16 (66.6)	0 (0.0)	2 (14.2)	0.0012 *
Nalidixic acid		44 (62.8)	15 (57.6)	20 (83.3)	1 (33.3)	8 (57.1)	0.056
Chloramphenicol		1 (1.4)	0 (0.0)	0 (0.0)	0	1 (5.8)	0.36
Category							
Resistant		12 (17.1)	8 (30.8)	2 (8.3)	0 (0.0)	2 (11.8)	0.12
MDR		24 (34.3)	8 (30.8)	4 (16.7)	2 (66.7) *	10 (58.8) *	0.02
XDR		34 (48.6)	10 (38.5)	18 (75.0) *	1 (33.3)	5 (29.4)	0.01

Resistant: resistant to ≤2 antibiotics categories; MDR, multidrug-resistant: resistant to ≥3 and ≤5 different categories of antibiotics; XDR, extensively drug-resistant: resistant to ≥6 different categories of antibiotics. Fisher’s exact test was used to investigate statistical significance. * *p* ˂ 0.05.

**Table 8:** Relation among antibiotic resistance of and cyclomodulin genes in strains isolated from patients with colon diseases.

	Total Strains CM^+^ *n* = 70 (%)	Cyclomodulins *n* = (%)									
		*pks*	*cnf*	*cdt*	*cif*	*cnf/cdt*	*cnf/cif*	*cdt/cif*	*cdt/pks*	*cnf/cdt/cif*	*p* Value
		*n =* 14 (%)	*n =* 10 (%)	*n =* 25 (%)	*n =* 3 (%)	*n =* 6 (%)	*n =* 4 (%)	*n =* 2 (%)	*n =* 2 (%)	*n =* 4 (%)	
Susceptible	0 (0.0)	0 (0.0)	0 (0.0)	0 (0.0)	0 (0.0)	0 (0.0)	0 (0.0)	0 (0.0)	0 (0.0)	0 (0.0)	-
Resistant to 1	3 (4.3)	0 (0.0)	0 (0.0)	1 (4.0)	1 (33.3)	1 (25.0)	0 (0.0)	0 (0.0)	0 (0.0)	0 (0.0)	0.31
Resistant to ˃2	67 (95.7) *	14 (100)	10 (100)	24 (96.0)	3 (66.6)	3 (75.0)	4 (100)	2 (100)	2 (100)	4 (100)	0.6

Fisher’s exact test was used to investigate statistical significance. * *p* ˂ 0.05 comparison among bacteria resistant to 1 antibiotic vs. resistant to ˃2.

## Data Availability

Data is contained within the article or Supplementary Materials.

## Supplementary Materials

The following are available online at https://www.mdpi.com/article/10.3390/pathogens11010014/s1, Table S1: Primers used in this study [45,46,47,48,49,50].
