# Supplementary material for: Prevalence of Cyclomodulin-Positive E. coli and Klebsiella spp. Strains in Mexican Patients with Colon Diseases and Antimicrobial Resistance
Source: Pathogens. 2021 Dec 23;11(1):14. doi: 10.3390/pathogens11010014 (PMC8779131; doi:10.3390/pathogens11010014)
Supplement: Supplementary file 1 [file pathogens-11-00014-s001.zip › pathogens-1478030-supplementary.pdf]

Supplementary Table S1. Primers used in this study

Bacteria	Primer	Sequence (5´-3´)	Size	Reference
E. coli	16S F	GTTAATACCTTTGCTCATTGA	340	[45]
	16S R	ACCAGGGTATCTAATCCTGTT		
	ChuA-F	GACGAACCAACGGTCAGGAT	279	[46]
	ChuA-R	TGCCGCCAGTACCAAAGACA		
	YjaA-F	TGAAGTGTCAGGAGACGCTG	211	
	YjaA-R	ATGGAGAATGCGTTCCTCAAC		
	TspE4C2.1-F	GAGTAATGTCGGGGCATTCA	152	
	TspE4C2.1-R	CGCGCCAACAAAGTATTACG		
	clbB-F	GCGCATCCTCAAGAGTAAATA	280	[25]
	clbB-R	GCGCTCTATGCTCATCAACC		
	CNF-1s	GGGGGAAGTACAGAAGAATTA	1112	[27]
	CNF-1as	TTGCCGTCCACTCTCACCAGT		
	CDT-s1	GAAAGTAAATGGAATATAAATGTCCG	555	[47]
	CDT-IIIasa	TTTGTGTCGGTGCAGCAGGGAAAA		
	cif-int-s	AACAGATGGCAACAGACTGG	383	[13]
	cif-int-as	AGTCAATGCTTTATGCGTCAT		
E. coli-EPEC	eae-F	TCAATGCAGTTCCGTTATCAGTT	482	[48]
	eae-R	GTA AAGTCCGTTACCCCAACCTG		
	bfp-F	GGA AGTCAAATTCATGGGGGTAT	300	
	bfp-R	GGAATCAGACGCAGACTGGTAGT		
E. coli-DAEC	daaE -F	GAACGT TGGTTAATGTGGGGTAA	542	[48]
	daaE -R	TATTCACCGGTCGGTTATCAGT		
Klebsiella	16S F	ACTCCTACGGGAGGCAGCAGT	383	[49]
	16S R	TATTACCGCGGCTGCTGGC		
	clbB-F	GCGCATCCTCAAGAGTAAATA	280	
	clbB-R	GCGCTCTATGCTCATCAACC		
Enterococcus faecalis	16S F	CCCTTATTGTTAGTTGCCATCATT	144	[50]
	16S R	ACTCGTTGTACTTCCCATTGT		

References

45. Malinen, E.; Kassinen, A.; Rinttila, T.; Palva, A. Comparison of real-time PCR with SYBR Green I or 5'-nuclease assays and dot-blot hybridization with rDNA-targeted oligonucleotide probes in quantification of selected faecal bacteria. *Microbiology (Reading)* **2003**, *149*, 269–277, doi:10.1099/mic.0.25975-0.
46. Clermont, O.; Bonacorsi, S.; Bingen, E. Rapid and simple determination of the Escherichia coli phylogenetic group. *Appl Environ Microbiol* **2000**, *66*, 4555–4558, doi:10.1128/aem.66.10.4555-4558.2000.
47. Toth, I.; Nougayrede, J.P.; Dobrindt, U.; Ledger, T.N.; Boury, M.; Morabito, S.; Fujiwara, T.; Sugai, M.; Hacker, J.; Oswald, E. Cytolethal distending toxin type I and type IV genes are framed with lambdoid prophage genes in extraintestinal pathogenic Escherichia coli. *Infect Immun* **2009**, *77*, 492–500, doi:10.1128/IAI.00962-08.
48. Vidal, M.; Kruger, E.; Duran, C.; Lagos, R.; Levine, M.; Prado, V.; Toro, C.; Vidal, R. Single multiplex PCR assay to identify simultaneously the six categories of diarrheagenic Escherichia coli associated with enteric infections. *J Clin Microbiol* **2005**, *43*, 5362–5365, doi:10.1128/JCM.43.10.5362-5365.2005.
49. Brisse, S.; Verhoef, J. Phylogenetic diversity of Klebsiella pneumoniae and Klebsiella oxytoca clinical isolates revealed by randomly amplified polymorphic DNA, gyrA and parC genes sequencing and automated ribotyping. *Int J Syst Evol Microbiol* **2001**, *51*, 915–924, doi:10.1099/00207713-51-3-915.
50. Rinttila, T.; Kassinen, A.; Malinen, E.; Krogius, L.; Palva, A. Development of an extensive set of 16S rDNA-targeted primers for quantification of pathogenic and indigenous bacteria in faecal samples by real-time PCR. *J Appl Microbiol* **2004**, *97*, 1166–1177, doi:10.1111/j.1365-2672.2004.02409.x.
